# Notes on the Medical History and Statistics of the British Legion in Spain

**Published:** 1838-04-01

**Authors:** 


### Notes on the Medical History and Statistics of the British Legion in Spain; comprising the Result of Gun-shot Wounds, in relation to important Questions in Surgery. By *Rutherford Alcock*, K.T.S. &c., Deputy Inspector-General of Hospitals, with the Auxiliary Forces in Portugal and Spain. Pp. 101.

We have perused the "Notes on the Medical History and Statistics of the British Legion in Spain" with no inconsiderable degree of interest, as well as pleasure—the former from the mass of useful information condensed in so small a space—the latter from the gratifying conviction that our professional brethren are not unmindful of the interests of the profession, although surrounded with difficulties, dangers, and privations of the most formidable description.

It is not less pleasing to us to observe that Mr. Alcock, in the very outset of his work, pays the tribute he considers due to the officers of the medical department of the Legion, for the zeal, ability, and steadiness of purpose with which they encountered dangers the most fearful, difficulties the most trying, and fulfilled the arduous duties imposed upon them with a perseverance alike creditable to their courage and honourable to their feelings—he says

"I feel there is another duty to perform—that of rendering justice to the zeal, ability, and courage, of a numerous medical staff, who entered the service regardless of the taunts and sneers at home, and were neither prevented by privation nor danger from discharging their duties faithfully, during a period abounding in both."

No stronger proof can be given of the danger to which the medical officers were exposed than the fact as stated by Mr. Alcock, that "seventeen" died during the period of service, and "fifteen of these in the first Winter."

The author has very properly divided his treatise into two parts—the first comprising the formation of the Legion,—its march to the interior of the northern provinces of Spain,—its heartrending sufferings and losses in Vittoria, Bribiesca, and Santander, and eventual return to the north coast—the second extending from that period to the termination of the service, wherein is detailed the most active and truly valuable portion of the services of the Legion, and at the same time furnishing the medical enquirer with an abundance of highly useful and instructive facts resulting from the treatment of fifteen hundred cases of gun-shot wounds.

With respect to the policy that led troops raw and undisciplined, as were those of the Legion, on the approach of Winter, to a distance from the coast, we have nothing to say beyond the expression of our conviction as medical men, that to this unnecessary, and to us unjustifiable act, is to be attributed in a great degree the last of miseries and misfortunes, of the worst description, that befell the troops, together with a sacrifice of life that is absolutely appalling, during the period of their service in Bribiesca and Vittoria. To this may be added the total disregard, on the part of the Spanish authorities of every principle of honour, humanity, and feeling, in withholding from the Legion the very necessaries of life, and sacrificing at the shrine of their jealousy and national hatred of all foreigners, not only the property of the government, but the lives of those who came to shed their blood in the ca use of their Sovereign.

In the Autumn of 1835, between seven and eight thousand recruits for the Legion were assembled on the north coast of Spain, at Santander, Bilbao, and San Sebastian. Of these about 3,500 were English, 2,800 Scotch, and 1,800 Irish—in addition to which, during the period of service, at different intervals, recruits were sent out probably amounting to 1,500. In the selection of these men, the most disgraceful and wanton abandonment of all rule was most pertinaciously persisted in throughout; and it becomes a matter of some difficulty to determine whether the agents in this country were the most to blame in the choice of men, or the Spanish authorities in Vitoria, for the utter neglect of everything that was requisite even for their very existence.

The author proceeds to notice the peculiarities, physical and moral, of the men of the three countries, from which he draws conclusions of the highest importance, both in a medical and military point of view—conclusions which, when fully established as general rules, must necessarily become of the first consequence to the Legislature. He observes—

"The English were upon the whole a bad class as to physical capacity—the same observation applies to the Scotch, who were chiefly from the manufacturing towns—the Irish were, undoubtedly, of all the Legion, the men who were physically and morally the best adapted for the service. It often seemed to me that both English and Scotch were quite capable of growing thin, miserable, and sick, upon the moody anticipation of evils and privations, while the reality itself seldom broke down the Irishman—and never until it produced actual disease."

In support of these opinions the author states that, during the first Winter at Vitoria, "one third nearly of the English brigade of infantry was swept into the hospitals with great rapidity—the Scotch next, about one fifth—and the Irish in a comparatively moderate proportion, probably not more than one eighth." It appears consequently from these statements that the Irish are, of all British troopts the best calculated to encounter privations and hardships, and to ward off, by their peculiar elasticity of animal spirits, diseases and dangers under which the less volatile part of our nation rapidly succumb.

During the period of service the total number of cases treated may be stated "in round numbers to be 14,000, and the deaths in hospital including wounded 2,000, making the proportion of deaths to cases treated one in seven, or of deaths to the whole force about one in four and two thirds."

The disease which thinned the ranks of the Legion in Vitoria appears to have been of a low typhoid character, some of the worst features of which, may, with much probability be referred to the wretched state of the men, as regarded clothing, bedding, and in short every comfort as well as necessary: the inferior quality of the provisions—their deficiency in quantity—and last, not least, the strong probability, that by far the greater portion of those who fell victims to the disease, were men of profligate and debauched habits, whose constitutions, already deteriorated, had no power to resist the debilitating and combined influence of privations and disease. These circumstances may in some degree, account for the vast number of cases of gangrene of the lower extremities supervening on the fever, which occurred in the hospitals of Vitoria.

"Although," observes Mr. Alcock, "exact data on the subject are wanting, I cannot be far from the truth in stating, that, I saw three hundred cases of gangrened extremities."

Having, with great difficulty obtained the Convent of San Antonio, as a surgical hospital, we are informed that—

"Here a great number of amputations were performed at the tarsal and metatarsal articulation, and across the metatarsal bones, also of both legs; for it was rare that one foot or leg alone was involved. These were necessarily performed under very unfavourable circumstances, on patients debilitated by fever and dysentery—of the result, I cannot however, give more than a return of those who recovered and were at Santander when I visited that depôt, although this number is not probably more than a fifth of the number of operations.

| | |
|---|---|
| Amputation of both legs . . . . . . . | 6 |
| Amputation of one leg . . . . . . . . | 1 |
| Partial amputation of both feet . . . . . | 2 |
| Partial amputation of one foot . . . . . | 3 |
| | 12" |

That so small a number of those operated upon, for gangrene of the lower extremities, recovered, can scarcely excite our wonder, on reflecting that all of these had passed through the ordeal of a disease which left its withering influence, for months after convalescence, even upon the most favoured and robust constitutions.

The usual course of the disease is thus described by our Author :—

"The patient felt for some days a general *sensation* of lassitude, frequent nausea or even vomiting, attended with pain of the head alone, or in others down the spine, sometimes extending to the whole frame. A disordered state of the bowels, diarrhœa more or less violent and frequent, very often was an attendant, though less prominent subject of complaint. No longer able to perform his duty, the patient would at last report himself; and this preliminary stage varied much in duration, from twenty-four hours to as many days, but more generally from two to five days. On examining the patient, the medical officer would often find a full pulse, flushed face, dry, hot skin, violent pain of the head, a crusted or loaded state of the tongue, with total prostration of strength; at other times greatly diminished vitality, purple and livid countenance, or skin shrunk. Delirium very quickly supervened. The tongue and fauces became dry and hardened, the lips loaded with sordes. Dysentery became developed, the rapid decline of the pulse and all the powers of life followed, and from the tenth to the twentieth day the patient died.

During this period the feet, partially or entirely, sometimes including the whole of the legs, would run rapidly through all the stages of gangrene. In others again, dysentery and its train of symptoms presented the leading feature with the same fever quickly supervening, running a still more rapid course."

From the disgracefully inefficient manner in which Mr. Alcock states the hospitals to have been supplied with stores of every description, and especially medical ones, it is not surprising that a mortality of between two and three hundred per month, for three months in succession, should have been the painful result;—but we turn with feelings of mingled sorrow and disgust from the contemplation of sufferings, heartrending beyond description, (sufferings, too, that derived their principal source from the perfidious conduct of those, whose interest it was, in every point of view, to have adopted a different course) to the second part of this eventful history, over the commencement of which, at all events, brighter prospects and better hopes appeared to dawn.

To the medical world this is unquestionably the most interesting and valuable part of the work, as many facts and results here authenticated, contribute in no slight degree towards the confirmation of some important points in military surgery.

The establishment of good and efficient hospitals at San Sebastian, together with their vicinity, generally, to the scenes of action, rendered the duties of the medical officers less painful than heretofore, and in some measure compensated the soldier, for the privations and miseries so unsparingly inflicted upon him, in the interior of the country.

The surgical hospital, part of the Convent San Telmo, capable on an emergency of containing eight hundred patients, appears to have been peculiarly well adapted to the purpose for which it was applied.

"The great thickness of the Convent walls assisted to keep the wards cool in Summer and warm in Winter; so that with order and cleanliness, which was strictly enforced, the whole hospital was perfectly free from all bad odour and the establishment was provided, after the first week or two of its formation, with diets of excellent quality and with every thing most necessary to the efficient treatment of the wounded and the regularity and good arrangement of a military hospital.

The patients were carefully classed according to their injuries—an arrangement, which the author very justly observes, he has "ever considered of great importance and productive of not less advantage to the patient than the surgeon."

Before noticing the results of the various actions we would most willingly, did our limits permit, enter fully upon the very valuable observations of Mr. Alcock in respect to the transport of wounded from the field, as well as the formation of a corps whose services should be especially devoted to the hospital department and independent of every other command, save the head of that branch of the service—but of this, as of very many other parts of these highly interesting "Notes," we are unable to give a brief and general outline, and we refer our readers to the work itself. The first action of any importance in which the troops of the Legion were engaged was that of the 5th of May, 1836, when an attack was made upon the triple lines of defence thrown up by the enemy in front of San Sebastian, and which was followed, at intervals, by others in the order given below. The returns from the 5th May do not commence before the 9th day—

"All very slight cases and a proportion of the most severe injuries having before that time disappeared, gives a total of 382—mortality 1 to $9\frac{11}{43}$.

Defence of the Lines on the 6th of June, 1836,—66—mortality 1 to $6\frac{3}{5}$.

Defence of the Lines 1st October, 1836—158—mortality nearly 1 to 6.

Several successive attacks on the entrenched Lines of the enemy during three days in March 1837, and the battle in position on the 16th—490—mortality 1 to 6.

Storming of Irun 16th and 17th of May, 1837—83—mortality 1 to $5\frac{3}{16}$

Wounded resulting from these and all minor affairs in Guipuscoa, from May, 1836 to June, 1837, admitted into hospital give a total of—1351—mortality nearly 1 to 7.

In considering the mortality in the different classes of wounds, beginning with gun-shot *injuries of the head* producing fracture, we find the general return gives 28 cases of fractured skull, of which 22 died, giving an average mortality of 1 to $11\frac{3}{11}$.

Scalp wounds—61—2 only died.

Penetrating wounds of thorax—38—two discharged to duty.

Penetrating wounds of both thorax and abdomen—3 occurred and all died.

Penetrating wounds of the abdomen from gun-shot, 19—one only recovered.

Penetrating wounds of joints—37 admitted into hospital—21 died.

Gun-shot fractures of the femur, 32—11 partial—21 complete.

Of the 11 partial—3 died.

Of the 21 complete—16 died.

Fifteen of the 21 were "reserved for treatment"—of these three cases of secondary hæmorrhage, for which, in two cases, amputation was performed—1 died—1 recovered—and in the third, the state of the patient forbade amputation, and the femoral artery was tied at its upper third; sphacelus of the foot supervened, and on the 10th day return of hæmorrhage and death. The artery and vein were found ulcerated. The ligature was applied about three quarters of an inch above the giving off of the profunda. The *channel of the lower portion was perfectly unobstructed*—proving as the author correctly observes—"the soundness of the principle which establishes the necessity of securing both ends of a wounded artery whenever it be possible."

The result of these cases reserved for treatment and the preparations shew

"How inadequate are even Nature's *vigorous* efforts to repair the mischief almost invariably the result of a gunshot fracture of the femur. These cases and the preparations resulting, are calculated to establish several other data of importance, viz:—

1st, That there is a relative and proportionate activity of the absorbent and secreting vessels established during the progress of cure.

2nd, That the different functions and actions brought into play by the fracture, in the bone and surrounding parts, particularly in the periosteum, have a constant and injurious tendency to extend far beyond the seat of injury, and the limits where their action would seem alone required or beneficial.

3rd, That loose fragments are readily cemented together, and do not necessarily, nor frequently become foreign bodies; consequently that no harassing operations should ever be performed to remove them.

Of gun-shot fractures of the leg, not complicated with injury to the joints, there were 57 cases, of which twenty died—1 to 2 $\frac{17}{20}$.

Of gun-shot fractures of the humerus not implicating joints, 31, of which 11 died—1 to 2 $\frac{2}{11}$.

There were 52 cases of gun-shot fracture of the hand and forearm, of which 3 died.

The general severe wounds amount to 403 of which 45 died; as nearly as may be, one ninth; nine of these from tetanus.

Out of 1,500 cases of men and officers, no case of secondary hemorrhage occurred in simple wounds without fracture. There were 12 cases, however, of secondary hemorrhage, many of which required ligatures."

Before concluding we shall briefly notice another most important question, namely—that of

"Delayed amputation—three periods have been defined for amputation, the

1st, Comprising the period between the receipt of the injury, and the appearance of the inflammatory symptoms.

2nd, When the inflammatory action has commenced and is more or less capable of disturbing the animal economy.

3rd, When the violence of the inflammatory symptoms and symptomatic fever have abated; that is, when the suppurative stage is fully established."

The author's remarks upon this question are exceedingly valuable and well worth the serious attention of all whose opportunities for observation and experiment enable them to throw light upon points of such vast importance in military surgery.

In conclusion, we cannot but lament the want of time and space to do ample justice to the talent and persevering industry of our author, whose "Notes," we feel assured, will be read with avidity by all who have the interest and welfare of the profession at heart. To those destined for the military part of the profession, it must ever remain an invaluable memorial of what *can* be done, even on active service, and act as a stimulus to others to follow in a path so ably marked out for them.

